# Lupus bulleux généralisé: présentation initiale rare du lupus érythémateux systémique

**DOI:** 10.11604/pamj.2013.16.56.3158

**Published:** 2013-10-16

**Authors:** Iman Hadj, Fatima Zahra Mernissi

**Affiliations:** 1 CHU Hassan II, Service de Dermatologie, Fès, Maroc

**Keywords:** Lupus bulleux, épidermolyse bulleuse, dermatoses bulleuses auto-immunes, bullous lupus, epidermolysis bullosa, autoimmune bullous dermatoses

## Image en médecine

Le lupus bulleux est rare, il représente moins de 5% des lupus. Il touche le sujet jeune de sexe féminin, il se manifeste par des bulles ou vésiculobulles souvent généralisées d'installation brutale, apparaissant sur peau érythémateuse ou saine, sur des zones photo et non photo-exposées, qui disparaissant sans cicatrice ni grain de milium. Il s'associe souvent à une atteinte systémique notamment rénale, les principaux diagnostics différentiels sont: l'épidermolyse bulleuse acquise, les autres dermatoses bulleuses auto-immunes, et les toxidermies bulleuses. Nous rapportons le cas d'un patient de 31 ans, sans antécédents notables, qui était hospitalisé pour des lésions bulleuses évoluant depuis 25 jours dans un contexte d'altération de l'état général, sans notion de photosensibilité. L'examen trouvait des placards urticariens au niveau du tronc, des membres et des plis surmontés de vésiculobulles groupées par endroit en rosette, ainsi que des érosions buccales. Le bilan objectivait un syndrome inflammatoire, lymphopénie, un sédiment urinaire actif, une protéinurie de 24h positive, une hypocomplémentémie, et une positivité des anticorps antinucléaires et anticorps anti DNA natif. L'histologie cutanée et l'immunofluorescence directe(IFD) étaient en faveur d'un lupus bulleux et la ponction biopsie rénale en faveur d'une néphropathie lupique stade III. Le diagnostic de lupus bulleux a été posé devant la présence de 5 critères de l'ARA, les lésions bulleuses, l'aspect histologique et la présence de la bande lupique à l'IFD. Vue l'atteinte systémique, un traitement par prédnisone, hydroxychloroquine, et bolus d'ENDOXAN était introduit avec bonne évolution.

**Figure 1:**
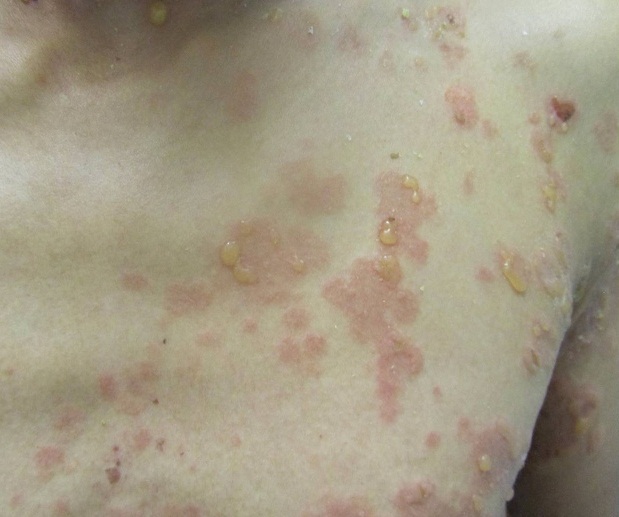
Placards urticariens du tronc surmontés de vésiculobulles

